# Magnitude of in-hospital mortality and its associated factors among patients undergone laparotomy at tertiary public hospitals, West Oromia, Ethiopia, 2022

**DOI:** 10.1186/s12893-024-02477-1

**Published:** 2024-06-20

**Authors:** Aliyi Benti Daba, Debrework Tesgera Beshah, Esayas Alemshet Tekletsadik

**Affiliations:** 1 Institute of health science, Wallaga University, Nekemte, Ethiopia; 2 College of Medicine and Health Science, University of Gondar, Gondar, Ethiopia

**Keywords:** In-hospital, Laparotomy, Magnitude, Mortality, Ethiopia

## Abstract

**Introduction:**

Laparotomy surgery, which involves making an incision in the abdominal cavity to treat serious abdominal disease and save the patient’s life, causes significant deaths in both developed and developing countries, including Ethiopia. The number studies examining in-hospital mortality rates among individuals that undergone laparotomy surgery and associated risk factors is limited.

**Objective:**

To assess the magnitude of in-hospital mortality and its associated factors among patients undergone laparotomy at tertiary hospitals, West Oromia, Ethiopia, 2022.

**Methods:**

An institutional based retrospective cross-sectional study was conducted from January 1, 2017, to December 31, 2021. Data were collected using systematic random sampling and based on structured and pretested abstraction sheets from 548 medical records and patient register log. Data were checked for completeness and consistency, coded, imported using Epi-data version 4.6, cleaned and analyzed using SPSS version 25 software. Variables with *p* < 0.2 in the Bi-variable logistic regression analysis were included in the multivariate logistic regression analysis. The fit of the model was checked by the Hosmer‒Lemeshow test. Using the odds ratio adjusted to 95% CI and a p value of 0.05, statistical significance was declared.

**Results:**

A total of 512 patient charts were reviewed, and the response rate was 93.43%. The overall magnitude of in-hospital mortality was 7.42% [95% CI: 5.4–9.8]. American society of Anesthesiology physiological status greater than III [AOR = 7.64 (95% CI: 3.12–18.66)], systolic blood pressure less than 90 mmHg [AOR = 6.11 (95% CI: 1.98–18.80)], preoperative sepsis [AOR = 3.54 (95% CI: 1.53–8.19)], ICU admission [AOR = 4.75 (95% CI: 1.50-14.96)], and total hospital stay greater than 14 days [(AOR = 6.76 (95% CI: 2.50-18.26)] were significantly associated with mortality after laparotomy surgery.

**Concussion:**

In this study, overall in- hospital mortality was high. Early identification patient’s American Society of Anesthesiologists physiological status and provision of early appropriate intervention, and pays special attention to patients admitted with low systolic blood pressure, preoperative sepsis, intensive care unit admission and prolonged hospital stay to improve patient outcomes after laparotomy surgery.

## Introduction

A laparotomy is a form of surgery requiring cutting into the abdominal cavity to treat serious abdominal diseases and save life [1]. It is the most common kind of surgery for conditions such as abdominal infections, intestinal obstruction, tumors, hernias, and intestinal traumas [2]. Globally, mortality rate for laparotomy surgery ranges between 8 and 18%, with varying rates in high, low, and middle-income countries [3–6].

Evidence from the Netherlands and Japan has shown that some patients recover and are discharged from the hospital after laparotomy surgery, while others develop an event that causes in-hospital mortality [7, 8]. A prior study revealed that the mortality rate after elective laparotomies in middle-income and low-income countries was equivalent to the death rate after emergency laparotomies in high-income countries [9]. Laparotomy is one of the top three surgical treatments in sub-Saharan Africa [10], accounts for a higher proportion of surgical procedures, and contributes to 2–3 times higher in-hospital mortality [11].

Evidence from Malawi and Tanzania showed that the magnitude of in-hospital mortality after laparotomy was 14.8% and 17%, respectively [12, 13]. A study from Ethiopia showed that the magnitude of in-hospital mortality after laparotomy was 8.1–8.5% ( [14, 15].

Preexisting comorbidities, systemic sepsis, inadequate tissue oxygenation, patients presenting late for surgery, delays prior to hospitalization, and late referral were all factors contributing to in-hospital mortality after laparotomy in Sub-Saharan Africa [16, 17]. Great progress has been made over the last two decades, and the Lancet Commission on Global Surgery 2030 recommends that district hospitals in both developed and developing countries provide basic surgical care and emergency care, including laparotomy, as well as a range of other services [18].

Ethiopia has worked to fill gaps in life-saving surgery through safe surgery, improving access to safe, necessary anaesthetic and surgical interventions in hospitals [19]. A previous study indicated that identifying factors associated with postoperative mortality was regarded as an indirect measure of surgical quality, aiding in the identification of reasons for increased mortality and the identification of selected practical intervention strategies [20, 21].

Despite the fact that several studies have been conducted in developed as well as developing countries among patients who had undergone laparotomy surgery, scholars are aware of very few studies on factors associated with in-hospital mortality among patients who had undergone laparotomy surgery. There have been no previous studies in the study area.

This multicentre study aims to assess magnitude of in-hospital mortality and associated factors for patients undergone laparotomy surgery in tertiary hospitals in western Oromia, Ethiopia, 2022.

## Methods

### Study area and period

The study was carried out from January 1, 2017, to December 31, 2021, at tertiary hospitals in western Oromia. Ambo University Referral Hospital, Nekemte Comprehensive Specialized Hospital, Wallaga University Referral Hospital, Jimma University Specialized Hospital, and Mettu Karl Comprehensive Specialized Hospital are the five public tertiary hospitals in Western Oromia. The study was conducted at the three hospitals randomly selected by lottery method and listed below. Ambo University Referral Hospital is located in Ambo, the capital of East Showa, and is 114 km from Ethiopia’s capital, Addis Ababa. The hospital serves around 5 million people and serves as a teaching hospital, offering multidisciplinary services, such as surgical interventions [22].

Nekemte Comprehensive Specialized Hospital is located in Nekemte town, East Wallaga, Ethiopia. The hospital provides services to approximately 5 million people in the catchment area and has multidisciplinary specialists [23, 24].

Mettu Karl Hospital is in Mettu, a city 600 km from Ethiopia’s capital, Addis Ababa. The hospital serves over 2.5 million people in the Ilu Ababor Region, Gambella Regional State, and the Southern Nationality and People’s Republic. The hospital provides multidisciplinary services, including surgery, and serves about 13,453 inpatients and 80,000 outpatients per year [25].

### Study design

An institutional-based cross-sectional study.

### Source and study population

#### Source population

Patients who undergone laparotomy surgery.

#### Study population

Patients who undergone laparotomy surgery from January 1, 2017- December 31, 2021 GC.

#### Inclusion and exclusion criteria

##### Inclusion criteria

Patients who undergone laparotomy surgery.

##### Exclusion criteria

Cesarean section, obstetrics & Gynecologic laparotomy.

### Study variables

#### Dependent variable

In-hospital mortality.

#### Independent variables

**Patient-related factors are** age, sex, ASA status, residence, presence of comorbidities, and systolic blood pressure.

**Disease-related factors are** initial admission, surgical indication or diagnosis, duration of onset of symptoms, urgency of surgery, presence of preoperative sepsis, and postoperative complications.

**Care-related factors are** preoperative imaging, laboratory tests, organ function tests, blood transfusion, intraoperative blood loss, preoperative medication, prophylaxis antibiotics, vasopressor use, anaesthesia drugs, use of the WHO checklist, duration of surgery, postoperative follow-up, hospital stay, intensive care unit admission, and duration of stay in the intensive care unit.

#### Operational definition

##### Laparotomy

Surgical incision into the abdominal cavity for treatment or diagnosis [1].

##### In-hospital mortality

Death occurred in the hospital after laparotomy [14].

##### ASA status

The American Society of Anaesthesiologists’ physiological status classification system categorises patients based on operative risk at the time of assessment into six classes (I-VI): ASA I: healthy patients; ASA II: mild systemic disease; ASA III: severe systemic disease with functional loss; ASA IV: severe systemic disease that is a constant threat to life; ASA V: morbid patients unlikely to survive with or without surgery; ASA VI: brain death and organ donation [26].

##### Comorbidity

Presence of any systemic disease other than surgical reason [14].

#### Sample size determination

The sample size (n) needed for the study was calculated according to an observational study performed at the University of Gondar Compressive Specialized Hospital [14] and determined by the single population proportion formula.

n = $$\frac{(\text{Z}\frac{{\upalpha }}{2}{)}^{2} \text{p}\left(1-p\right)}{{\text{d}}^{2}}$$ where

n = desired sample size.

P = the overall prevalence of mortality 8.1% [14].

$\text{Z}\frac{{\upalpha }}{2}$= critical value at the 95% confidence interval, i.e., 1.96

d = Margin of error between the sample and the proportion, i.e., 3% (0.03).

n = $\frac{{\left(1.96\right)}^{2} 0.081\left(1-0.081\right)}{{\left(0.03\right)}^{2}}$= 318, by adding a 10% =32 nonresponse rate sample size for the first objective = 350.

For the second objective, the sample size was calculated by considering significantly associated factors. Preoperative sepsis (AOR = 6.7), mechanism of abdominal injury (blunt abdominal injury) (AOR = 7.25), age > 65 (AOR = 6.7), and SBP < 90 mmHg (AOR = 8.6).

The final sample size for this study was taken from the 2nd objective: **332 + 33 (10%)** = **365**. Because the sampling procedure has a design effect (**1.5**), multiplied by the final sample size for the second objective, it was **365 × 1.5** = **548** (Table 1:).

**Table 1:** Sample size determination to assess the magnitude of in-hospital mortality and its associated factors among patients undergone laparotomy at tertiary hospitals, West Oromia, Ethiopia, 2022 (for second objective)

Variables		Proportion		OR	Two-sided confidence level	Power	Sample size
Associated factors mortality	Age > 65years (20/49)	P1	0.408	9.6	95	80	40(ref. [14])
	Reference (22/469)	P1	0.046				
	Presence of pre-op sepsis	P1	0.19%	6.7	95	80	90 (ref. [14])
	Reference/unexposed	P2	0.0125%				
	Mechanism of injury/Blunt (7/49)	P1	0.143%	7.25	95	80	332(ref. [15])
	Reference/penetrating(4/80)	P2	0.05%				
	SBP< 90mmmHg(8/24)	P1	0. 33%	8.6	95	80	50 (ref. [15])

### Sampling procedure

**Fig. 1:**
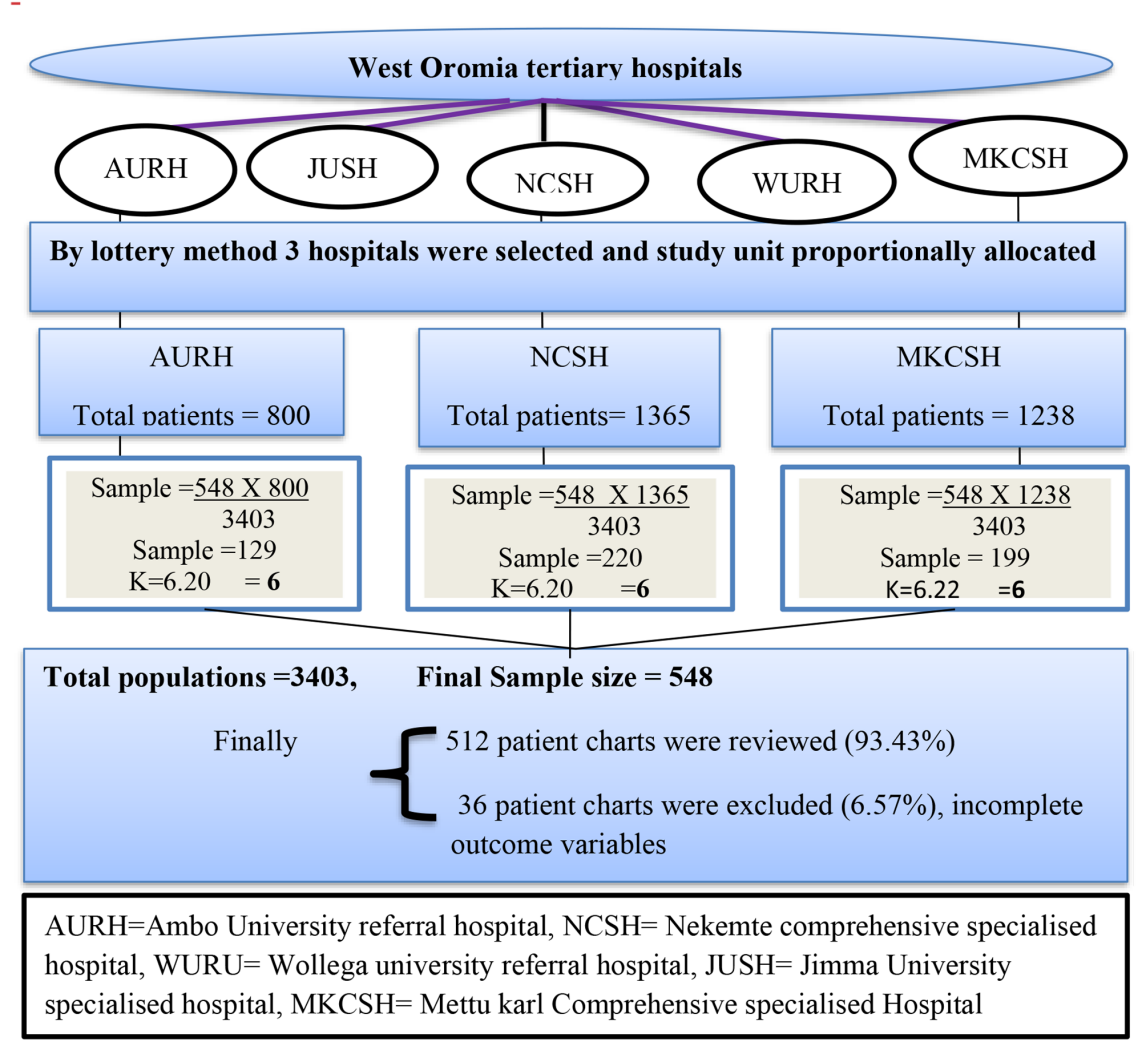
Schematic presentation of sampling procedure on magnitude of in-hospital mortality and its associated factors mortality among patients undergone laparotomy at tertiary hospitals, West Oromia, Ethiopia, 2022

All patients who undergone laparotomy surgery from January 1, 2017, to December 31, 2021, were included through a systematic random sampling technique using the hospital ward registration list as a sampling frame, and the sample size for selected hospitals was proportionally allocated (Fig. 1:).

### Data collection tools and procedure

A structured checklist prepared in English and adapted from [14, 27–29] was used to collect data. Data collectors collected all the information from the chart into a data abstraction format. Six BSc nurses (two in each hospital) were recruited to collect data and three MSC nurses (one in each hospital) to supervise daily activities in each hospital.

### Data quality management

Investigators gave training on the use of the study protocol to data collectors and supervisors. The abstract sheet was pretested on 27 (5%) medical records at the Wallaga University Referral Hospital. The reliability of the data extraction sheet has been checked and verified by experts. During data collection, all data were collected and correctly compiled in a prepared format. The investigators supervised and facilitated the entire data collection and completeness check process.

### Data processing and analysis procedure

The data were checked for completeness and consistency and then coded and entered using Epi-Data Version 4.6. The data were also cleaned and analyzed using SPSS version 25. The outliers were screened through visual assessment for scattered plot diagrams. Box plots and histograms, as well as Kolmogorov-Spiro, were used to test normality. Descriptive statistics were computed to determine frequencies and summary statistics (mean, standard deviation, median, IQR, and percentage). The data were summarized and presented using text, tables, and charts. All variables with *P* < 0.2 in the bi-variable analysis were included in the final model of multivariable analysis to control possible confounders. The correlation between independent variables was tested for multicollinearity by using the variance inflation factor (VIF = 1.17–4.23) and correlation matrix < 1. Model fitness was checked with the Hosmer-Lemeshow test (0.103). A statistically significant value was declared using the adjusted odds ratio (AOR) at a 95% confidence interval and a p value < 0.05.

## Results

### Patient-related factors

A total of 512 patient charts were reviewed with a 93.43% response rate, while 36 (6.57%) were excluded due to missing or incomplete outcome variables. The majority of them, 401 (78.32%), were between the ages of 18 and 64 year. Patients ranged in age from 1 to 92 year, with a mean of 36.24 and SD = 0.755 year. Among the 512 study participants, 319 (62.3%) were males, and 281 (54.9%) were from rural areas. The majority of patients (*n* = 388, or 75.8%) had American Society of Anesthesiologists Physiological Status I. Preoperatively associated comorbidities were present in 73 (14.3%) of the patients. The majority of the 482 study participants (94.1%) had a systolic blood pressure of greater than 90 mmHg (Table 2).

**Table 2:** Patients related factors among patients undergone laparotomy at tertiary hospitals, West Oromia, Ethiopia, 2022 (*n* = 512)

Variables	Category	Frequency	Percentage
Age	< 18yrs.	52	10.16
	18–64 yrs.	401	78.32
	≥ 65 yrs.	59	11.52
Sex	Male	319	62.3%
	Female	193	37.7%
Residence	Urban	231	45.1%
	Rural	281	54.9%
ASA status	ASA class I	388	75.8%
	ASA class II	58	11.3%
	ASA class III	36	7.0%
	ASA class Iv	30	5.9%
Comorbidity status	Yes	73	14.3%
	No	439	85.7%
Types of comorbidity (*n* = 73)	Hypertension	36	49.31%
	Diabetes mellitus	13	17.80%
	HIV/AIDS	6	8.21%
	Asthma	18	24.65%
Pre-operative Systolic Blood Pressure	< 90mmhg	30	5.9%
	≥ 90mmhg	482	94.1%

Note: ASA- American Society of Anaesthesiology physical status, HIV/AIDS-human immune deficiency virus /Acquired immune deficiency syndrome

### Disease-related factors

Among the 512 participants, 294 (57.4%) were seen initially at the emergency department, and 82 (16%) were admitted for elective laparotomy. The majority of patients, 482 (82.2%), presented within 7 days of their initial symptoms, and 115 (22.5%) had preoperative sepsis. Thirty (36.6%) patients were admitted for elective laparotomy for cholelithiasis; fourteen (17.1%) had gastric outlet obstruction; and eleven (13.4%) had rectal cancer. The majority of patients (84.9%) were admitted for a non-traumatic emergency laparotomy. The most common reason for a non-traumatic emergency laparotomy was to diagnose acute appendicitis, which accounted for 27.55%, followed by small bowel obstruction, which accounted for 17.8%. The most common reason for a traumatic emergency laparotomy was diaphragmatic injury 29.6% (Table 3):.

**Table 3:** Admission status of patients undergone laparotomy at tertiary hospitals, West Oromia, Ethiopia, 2022 (*n* = 512)

Variables	Category	Frequency	Percentage
The Initial route of admission	Emergency department	294	57.4%
	Referred from Health centres’	122	23.8%
	Transferred from wards	67	13.1%
	Direct admission from private clinic	29	5.7%
Duration of symptoms	≤ 7 days	423	82.6%
	> 7 days	89	17.4%
Preoperative sepsis	Yes	115	22.5%
	No	397	77.5%
Type of surgery	Elective	82	16%
	Emergency	430	84%
	Abdominal benign tumour	5	6.1%
Elective laparotomy	Adenocarcinoma Colon	6	7.3%
	Rectal Cancer	11	13.4%
	Gastric Cancer	1	1.2%
	Colostomy closure	8	9.8%
	Cholelithiasis	30	36.6%
	Gastric outlet obstruction	14	17.1%
	Sigmoid volvulus	7	8.5%
If due to malignancy, severity	None	4	22.2%
	Nodal-metastases	10	55.6%
	Primary only	4	22.2%
Indication of emergency laparotomy	Non traumatic	365	84.9%
	Traumatic	65	15.1%
Specific indication non-traumatic emergency laparotomy (*n* = 365)	Peritonitis	37	7.2%
	Perforation peptic ulcer disease	15	2.9%
	Small bowel obstruction	61	11.9%
	Large bowel obstruction	64	12.5%
	Appendicitis	141	27.5%
	Cholecystitis	17	3.3%
	Adhesion	10	2.0%
	Others*	20	5.6%
Mechanism of injury (*n* = 65)	Blunt	10	15.4%
	Penetrating	55	84.6%
Isolated organ injury(*n* = 65)	Small bowel	9	13.8%
	Spleen	3	4.6%
	Large Bowel	8	12.3%
	Stomach	7	10.8%
	Retro Peritoneal Haemorrhage	5	7.7%
	Diaphragmatic injury	29	44.6%
	Abdominal-thoracic injury	4	6.2%

*other includes: strangulated hernia, Abdominal wound dehiscence, Colitis, Haemorrhage, Ischemia, Anastomotic leak, abdominal abscess

### Health care intervention

An abdominal ultrasound was performed for 43.9% of the 512 patients. Among 512 patients, 73.4% had hemoglobin 11–18 mg/dl, 93.4% of study participants had a platelet count of 150–450 × 109/L, and 73.4% of study participants had a platelet count of 4–11 × 103/mm^3^. Antibiotic prophylaxis was administered to 100% of patients, but only 86.5% received preoperative medication. Within 30 days, 3.3% of patients had a re-laparotomy within 30 days (Table 4:).

**Table 4:** Factors related to health care intervention among patients undergone laparotomy surgery at tertiary hospitals, West Oromia, Ethiopia, 2022 (*n* = 512)

Variables	Category	Frequency	Percentage
Pre-operative-imaging modalities	Computer Tomography	12	2.3%
	X-ray	219	42.8%
	Ultra Sound	225	43.9%
	No imaging done	56	10.9%
Operation within one month including this procedure	One time only	495	96.7%
	Two times	17	3.3%
Laboratory investigation	Yes	512	100%
Haemoglobin (g/dl)	< 11 mg/dl	90	17.6%
	11–18 mg/dl	376	73.4%
	> 18 mg/dl	46	9.0%
Platelets counts	< 150 × 10^9^/L	17	3.3%
	150–450 × 10^9^/L	462	90.3%
	> 450 × 10^9^/L	33	6.4%
White blood cell count	< 4 × 10^3^/mm3	20	3.9%
	4–11 × 10^3^/mm3	376	73.4%
	> 11 × 10^3^/mm3	116	22.7%
Organ function test	Yes	6	1.2%
	No	506	98.8%
Blood transfusion	Yes	79	15.4%
	No	433	84.6%
Pre-operative medication	Yes	443	86.5%
	No	69	13.5%

#### Surgical procedure

Appendectomy was the most common surgical procedure performed (27.1%), followed by anastomosis resection (15.2%). Almost all patients (99.8%) undergone surgery under general anesthesia, and the WHO surgical safety checklist was used for 86.3% of the patients. One hundred and seven (20.9%) patients received early surgical intervention, 79.1% received surgical intervention more than six hours after hospital admission, and the majority of patients (90%) experienced intraoperative blood loss of ≤ 500 ml (Table 5).

**Table 5:** Surgical procedure among patients undergone laparotomy surgery at tertiary hospitals, West Oromia, Ethiopia, 2022 (*n* = 512)

Variables	Category	Frequency	Percentage
Consciousness-recovery from anaesthesia	Fully-awake	448	87.5%
	Half-awake	62	12.1%
	Not awake	2	0.4%
Duration of anaesthesia	≤ 2 h	465	90.8%
	> 2 h	47	9.2%
Main procedure	Repair of perforation peptic ulcer disease	14	2.7%
	Gastrojejunostomy	15	2.9%
	Abdominal wall closure	9	1.8%
	Small bowel resection	71	13.9%
	Colorectal resection	19	3.7%
	Exploratory laparotomy	47	9.2%
	Re-sectional anastomosis	78	15.2%
	Appendectomy	141	27.5%
	Cholecystectomy	46	9.0%
	Diaphragmatic repair	18	3.6%
	Grams patch procedure	36	7.0%
	Others	18	3.6%
Used vasopressor/ inotrope?	Yes	24	4.7%
	No	488	95.3%
Use of WHO surgical safety checklist	Yes	442	86.3%
	No	70	13.7%
Time from admission to operation	< 6 h	107	20.9
	6:01–11:59 h	227	44.3
	12–23:59 h	74	14.5
	≥ 24 h	104	20.3
Duration of surgery (in hours)	≤ 2 h	490	95.7%
	> 2 h	22	4.3%
Intraoperative blood loss (ml)	≤ 500 ml	461	90%
	> 500 ml	51	10%

(*Other: Subtotal Gastrectomy, Adhesiolysis, Colectomy subtotal, Homeostasis, Gastric surgery, Drainage of abscess)*

#### Patient condition and transfer after surgery

**Fig. 2:**
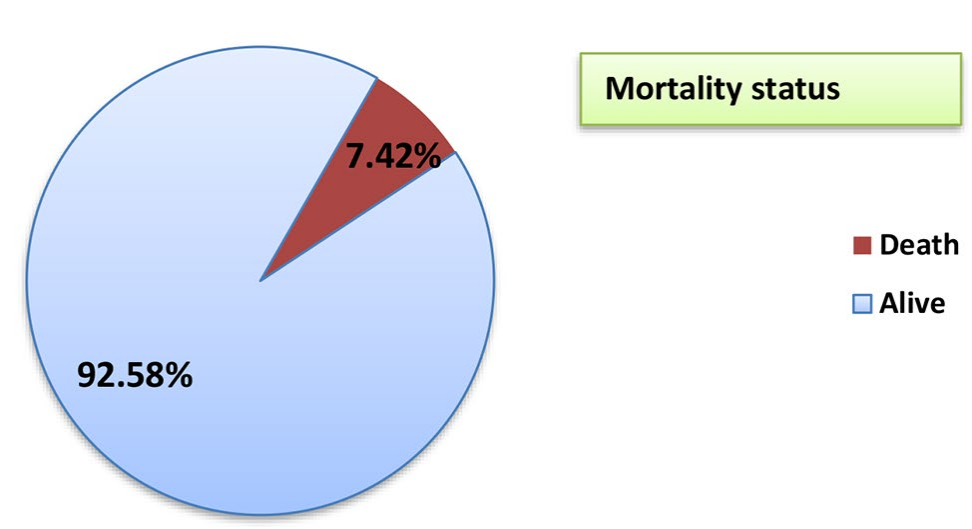
Discharge status. A total of 512 patients undergone laparotomy, the overall in-hospital mortality rate was 7.42%

Only 3.2% of patients were directly transferred from the operating theatre to the intensive care unit, while 94.3% of patients were transferred to the surgical ward after the operation. The majority of patients (92.2%) stayed in the hospital for less than 14 days, with a median of 7 and an interquartile range of 7 ± 4 days. One hundred fifty-one (29.5%) had postoperative surgical complications, including hemorrhage (3.3%), hospital-acquired pneumonia (2.9%), and wound infection (9.0%) (Table 6:). More than three quarters of patient’s undergone laparotomy, (92.58%) were discharged with alive (after improvement) (Fig. 2:).

**Table 6:** Post-operative care and hospital stay among patients undergone laparotomy surgery at tertiary hospitals, West Oromia, Ethiopia, 2022 (*n* = 512)

Variables	Category	Frequency	Percentage
Post-operative care following surgery	Surgical Ward	483	94.33%
	PACU	11	2.14%
	ICU	16	3.13%
	Died before discharge from OR	2	0.4%
ICU admission	Yes	30	5.9%
	No	482	94.1%
Length of post-op ICU stay(days)	≤ 5days	23	76.7%
	> 5 days	7	23.3%
The overall length of hospital stay	< 14 days	472	92.2%
	≥ 14 days	40	7.8%
Presence of postoperative complications	Yes	151	29.5%
	No	361	70.5%
Types of post-operative complication	Haemorrhage	17	11.2%
	Hospital acquired pneumonia	15	9.9%
	Intraabdominal complication	23	15.2%
	Wound site infection	46	30.0%
	Others	51	33.7%
Status at discharge	Death	38	7.42%
	Alive	474	92.58%

*(Others: Vomiting, postoperative ileus, sepsis, Evisceration, wound dehiscence)*

*PACU-Post Anaesthesia Care Unit, ICU: Intensive Care Unit, OR: Operation room*

### Factors associated with in-hospital mortality

In the final multivariable analysis, age of patients, ASA status, comorbidity, low systolic blood pressure at admission, presence of preoperative sepsis, re-laparotomy within 30 days, blood transfusion, duration of anesthesia, duration of surgery, intraoperative blood loss, ICU admission, intraoperative vasopressor use, length of hospital stay, and postoperative surgical complications had values of 0.2. A significant association between the outcome variable and multivariable logistic regression was discovered. Patients with ASA status III, low systolic blood pressure at admission, preoperative sepsis, ICU admission, and length of hospital stay of 14 had a p value of less than 0.05.

The odds of death after laparotomy surgery for patients with an ASA status of ≥ III was approximately 7.6 times higher than for patients with an ASA status of I and II [AOR = 7.64 (95% CI: 3.12–18.66)]. The odds of death after laparotomy was six times higher in patients with systolic blood pressures of less than 90 mmHg at admission [AOR = 6.11 (95% CI: 1.98–18.80)] than in patients with blood pressures of greater than 90 mmHg at admission. The odds of mortality after laparotomy surgery was 3.5 times higher in patients with preoperative sepsis than in patients without sepsis [AOR = 3.54 (95% CI: 1.53–8.19)]. Patients admitted to the intensive care unit had a 4.7 more likely to die than those who were not admitted. The odds of death among patients who stayed in the hospital for ≥ 14 days were 6.7 times greater than those who stayed for < 14 days [AOR = 6.76 (95% CI: 2.50–18.26)] (Table 7:).

**Table 7:** Bi-variable & multivariable logistic regression analysis on factors associated with in-hospital mortality after laparotomy (*n* = 512)

Variables	Category	Mortality status		COR(95% CI)	AOR (95% CI)	P value
		Death(n)	Alive(n)			
Age	≥ 65 yrs.	11	48	2.15 (0.69–6.67)	1.28 (0.25–6.54)	0.761
	18-64yrs.	22	379	0.546 (0.2–1.50)	0.54 (0.14–2.07)	0.371
	< 18 yrs.	5	47	1	1	
ASA status	III&IV	19	47	9.08(4.49–18.36)	7.64(3.12–18.66)**	**0.001**
	I&II	19	427	1	1	
Comorbidity	Yes	18	55	6.85 (3.41–13.75)	2.05 (0.21–19.43)	0.530
	No	20	419	1	1	
Preoperative SBP	< 90 mmHg	7	23	4.42(1.76–11.12)	6.11(1.98–18.80)**	**0.002**
	≥ 90 mmHg	31	451	1	1	
Preoperative Sepsis	Yes	18	97	3.49 (1.78–6.86)	3.54 (1.53–8.19)**	**0.003**
	No	20	377	1	1	
Re-laparotomy within30 days	2 times	5	12	5.83(1.93–17.54)	1.117(0.16–7.40)	0. 907
	1 time	33	463	1	1	
Blood transfusion	Yes	10	69	2.096(0.97–4.50)	0.68 (0.20–2.34)	0. 548
	No	28	405	1	1	
Anesthesia duration	> 2 h	8	39	2.97 (1.27–6.93)	1.53(0.42–5.56)	0.512
	≤ 2 h	30	435	1	1	
Vasopressor used	Yes	5	19	3.62(1.27–10.33)	1.22(0.26–5.70)	0.797
	No	33	455	1	1	
Duration of surgery	> 2 h	5	17	4.07 (1.41–11.73)	2.81(0.81–9.73)	0.102
	≤ 2 h	33	457	1	1	
Operative Blood loss	> 500 ml	13	38	5.96 (2.82–12.60)	2.5 (0.85–7.31)	0.093
	≤ 500 ml	25	436	1	1	
ICU admission	Yes	13	17	13.97(6.11–31.95)	4.75(1.50-14.96)*	**0. 008**
	No	25	457	1	1	
Hospital stay	≥ 14 days	16	24	13.63(6.35–29.26)	6.76(2.50-18.26)**	**0.001**
	< 14 days	22	450	1	1	
Postoperative Complication	Yes	26	125	4.78 (2.40–9.54)	1.46(0.565–3.78)	0.434
	No	12	349	1	1	

*** shows < 0.001 and ** <0.01

## Discussion

The overall in-hospital mortality rate among patients who had undergone laparotomy was 7.42% [95% CI: 5.4–9.8]. This study agreed with the studies performed at the University of Gondar Comprehensive Specialty Hospital [14], St. Paul Millennium Hospital, Addis Ababa, 8.5% [15], Nigeria, 8% [30]. These studies also show lower results than studies conducted in Mali (14.8%) [12], Ghana (11.5%) [31], Tanzania (17%) [27] and Denmark (20.2%) [32]. Possible explanations for the discrepancy include study duration, indication, and study design. The Mali study was conducted prospectively at a single center over six months among adults hospitalized for emergency laparotomies, whereas the Ghana and Tanzania studies included a one-year retrospective for emergency laparotomies. A study in Denmark was conducted among 18-year-old patients who were hospitalized for abdominal emergencies. The deviations may be due to differences in the respondents’ sociodemographic characteristics and the length of the study. The evidence also suggested that the study’s short duration may not have been long enough to adequately represent the full spectrum of mortality [28]. This study also compared less with two previous studies conducted in the United States, where mortality rates were 21% and 23.3% [33, 34]. The difference could be explained by the fact that both of these studies were conducted in a trauma center among patients undergoing traumatic laparotomies. There are also differences in age between study units. The first study, conducted in the United States, included only patients aged 16 and above, while the second study included patients aged 55 and above. Previous research found that when study units were elderly, the risk of death after laparotomy surgery increased [12, 35].

Regarding factors, patients with ASA status ≥ III were nearly eight times more likely to die than those with ASA status I and II. This study consistent with the studies performed at the University of Gondar Comprehensive Specialty Hospital [14]. In contrast, Addis Ababa, St. In the Paul Millennium Hospital study, patients’ ASA status had no effect on mortality after emergency laparotomy [15], and a study performed in South Africa found that patients with lower ASA had a higher mortality rate [28]. The difference could be explained by the study’s short duration and the age groups represented in the study units. The majority of study units in Addis Ababa study were found in the 19–29 age groups among patients with abdominal trauma, whereas the study in South Africa included adult patients who were admitted to emergency laparotomy surgery for only three months. A previous study discovered that older patients had a higher ASA physical status, which was associated with an increased incidence of postoperative adverse events. ASA may help improve surgical intervention by assessing patient age and physical condition [36]. The findings from this study are supported by studies done in Malawi [12], the UK [37–39], Singapore [40], the USA [33], Turkey [41] and Ireland [42]. The possible explanation for this consistency is that patients’ ASA status contributes to decreased physiological reserve and that the negative effect of underlying abdominal pathology may contribute to an increased risk of postoperative mortality in laparotomy patients [43].

In this study, patients with a SBP less than 90 mmHg at admission were approximately six times more likely to die than patients with a systolic blood pressure greater than 90 mmHg. This finding is supported by studies conducted in Gondar [14], Addis Ababa [15], the United States of America [34] and the Netherlands [7]. The reason for consistency may be explained by the possibility that patients with intra-abdominal disease may experience hypovolemia due to intraluminal fluid accumulation, intra-abdominal hemorrhage, distributive shock from severe inflammation, or hypovolemia from the gastrointestinal tract through vomiting or diarrhea. In these cases, fluid resuscitation will need to continue during the perioperative period in accordance with the underlying cause [44].

The current study identified that patients who had preoperative sepsis were nearly four times more likely to die than those who did not have preoperative sepsis. This finding is consistent with findings from a study performed at the University of Gondar Comprehensive specialized Hospital [14], UK and Brazil [37, 45]. Evidence from the USA also showed that the presence of severe sepsis was independently associated with mortality after laparotomy surgery [46]. It can be explained that patients with preoperative sepsis are more likely to develop postoperative complications that result in organ dysfunction due to tissue hypo-perfusion, increased morbidity and mortality, and hospitalizations costs [47–49].

In this study, intensive care unit admission was a significant factor associated with mortality. Patients admitted to the intensive care unit while in the hospital were nearly five times more likely to die than those who were not admitted. This finding is supported by research conducted in various parts of the world, including South Africa [28], Rwanda [50], the UK [39], Singapore [40], Ireland [42], the Netherlands [7], and China [51].

This may be explained by patients admitted to the intensive care unit having a poor clinical outcome following laparotomy surgery due to poor general health and multiple potential risk factors. The length of stay in the intensive care unit can have an impact on patient mortality. Early discharge from intensive care may save the patient’s life by preventing treatment and hospital complications [52].

The length of hospital stay was found to be significantly associated to mortality in this study. Patients who stayed in the hospital for more than 14 days were nearly seven times more likely to die than those who did not. This study is supported by research from South Africa and Nigeria that shows that patients hospitalized for more than 14 days after laparotomy surgery have a higher risk of dying. The length of hospital stay may reflect the severity of the disease, necessitating a lengthy hospital stay until recovery, or the patient’s medical condition [28, 53]. One possible explanation for the similarity is that patients who stay in the hospital for an extended period of time are more vulnerable to postoperative complications, nosocomial infections, and increased mortality after laparotomy surgery [54–56].

### Limitation and strength of the study

This is the first multicenter study conducted on in-hospital mortality after laparotomy at tertiary hospitals in the study area and Ethiopia, which may provide insight into the severity of the current problem and the need for the patient’s perioperative care and may be used as a reference for future researchers.

## Conclusion

In this study, the overall magnitude of in-hospital mortality after laparotomy was high. American Society of Anesthesiology physiological status, low systolic blood pressure at admission, presence of preoperative sepsis at admission, intensive care unit admission, and length of hospital stay greater than fourteen days were factors associated with in-hospital mortality among patients who undergone laparotomy surgery.

### Recommendations

#### To the respective institutions, hospitals, and health care professionals

General recommendation to decrease in-hospital mortality after laparotomy surgery.

- The authors’ advice is to use preoperative risk identification guidelines, appropriate perioperative resuscitation and optimisation, early control of the sources of sepsis, and appropriate monitoring in the postoperative critical care unit for patients who stay in the hospital for a long time.
- For seriously ill patients with sepsis, treatments, including fluid resuscitation, broad-spectrum antibiotics, and laboratory investigation, should be immediately initiated.
- For future researchers, we recommend that researchers who are interested in the area conduct prospective follow-up studies.

## Data Availability

The datasets used or analyzed during the current study are available from the corresponding author upon reasonable request through email: alsanbenti4760@gmail.com.

## Abbreviations

AOR: Adjusted Odd Ratio
ASA: American Society of Anesthesiologists
CI: Confidence Interval
ICU: Intensive Care Unit
SBP: Systolic Blood Pressure
UK: United Kingdom
USA: United States of America
WHO: World Health Organization

## References

[1] Strik C, Stommel MW, Schipper LJ, van Goor H, Ten Broek RP (2016). Risk factors for future repeat abdominal surgery. Langenbeck’s Archives Surg.

[2] Gejoe G, Yadev I, Rahul M (2017). Emergency laparotomies at a tertiary care center—a hospital-based cross-sectional study. Indian J Surg.

[3] Aggarwal G, Peden CJ, Mohammed MA, Pullyblank A, Williams B, Stephens T (2019). Evaluation of the collaborative use of an evidence-based care bundle in emergency laparotomy. JAMA Surg.

[4] Barazanchi AW, Xia W, MacFater W, Bhat S, MacFater H, Taneja A (2020). Risk factors for mortality after emergency laparotomy: scoping systematic review. ANZ J Surg.

[5] Stevens CL, Brown C, Watters DA (2018). Measuring outcomes of clinical care: victorian emergency laparotomy audit using quality investigator. World J Surg.

[6] Tolstrup M-B, Watt SK, Gögenur I (2017). Morbidity and mortality rates after emergency abdominal surgery: an analysis of 4346 patients scheduled for emergency laparotomy or laparoscopy. Langenbeck’s Archives Surg.

[7] Hietbrink F, Smeeing D, Karhof S, Jonkers HF, Houwert M, van Wessem K (2019). Outcome of trauma-related emergency laparotomies, in an era of far-reaching specialization. World J Emerg Surg.

[8] Shimada H, Fukagawa T, Haga Y, Oba K (2017). Does postoperative morbidity worsen the oncological outcome after radical surgery for gastrointestinal cancers? A systematic review of the literature. Annals Gastroenterological Surg.

[9] Pooled analysis of WHO Surgical (2019). Safety Checklist use and mortality after emergency laparotomy. J Br Surg.

[10] Watters D, Wilson L (2021). The comparability and utility of perioperative mortality rates in global health. Curr Anesthesiology Rep.

[11] Collaborative G. Mortality of emergency abdominal surgery in high-, middle-and low-income countries. 2016. 10.1002/bjs.10151 27145169

[12] Sincavage J, Msosa VJ, Katete C, Purcell LN, Charles A (2021). Postoperative complications and risk of Mortality after Laparotomy in a Resource-Limited setting. J Surg Res.

[13] Mbabala D. Predictors and short term outcomes for patients who underwent laparotomy at Muhimbili National Hospital a cross section study. Muhimbili University of Health and Allied Sciences; 2020.

[14] Oumer KE, Ahmed SA, Tawuye HY, Ferede YA (2021). Outcomes and associated factors among patients undergone emergency laparotomy: a retrospective study. Int J Surg Open.

[15] Abebe K, Bekele M, Tsehaye A, Lemmu B, Abebe E. Laparotomy for abdominal injury indication & outcome of patients at a teaching hospital in Addis Ababa, Ethiopia. Ethiop J Health Sci. 2019;29(4). 10.4314/ejhs.v29i4.12 PMC6689702 31447524

[16] Ogbuanya AU-O, Amah D (2018). Delay in presentation and challenges of treatment of complicated abdominal wall hernias in rural southeast Nigeria. Nigerian J Surg Sci.

[17] Ackland G, Abbott T, Cain D, Edwards M, Sultan P, Karmali S (2019). Preoperative systemic inflammation and perioperative myocardial injury: prospective observational multicentre cohort study of patients undergoing non-cardiac surgery. Br J Anaesth.

[18] Meara JG, Leather AJ, Hagander L, Alkire BC, Alonso N, Ameh EA (2015). Global surgery 2030: evidence and solutions for achieving health, welfare, and economic development. Lancet.

[19] Burssa D, Teshome A, Iverson K, Ahearn O, Ashengo T, Barash D (2017). Safe surgery for all: early lessons from implementing a national government-driven surgical plan in Ethiopia. World J Surg.

[20] Mortality of emergency (2016). Abdominal surgery in high-, middle- and low-income countries. Br J Surg.

[21] Krishnamurthy V, Ishwaraprasad G, Rajanna B, Samudyatha U, Pruthvik B (2016). Mortality pattern and trends in surgery wards: a five year retrospective study at a teaching hospital in Hassan district, Karnataka, India. Int Surg J.

[22] Kefale B, Betero G, Temesgen G, Degu A. Management practice, and treatment outcome and its associated factors among hospitalized stroke patient at Ambo University Referral Hospital, Ethiopia: an institutional Based Cross Sectional Study. 2019.

[23] Fetensa G, Milkiyas N, Besho M, Hasen T, Teshoma M. Assessment of knowledge and practice of life style modification among hypertensive patients at Nekemte specialized hospital, western Oromia, Ethiopia: a cross-sectional study design. J Cardiovasc Dis Diagnosis. 2019;7(6).

[24] Firdisa G, Tilahun T, Kejela G (2022). Determinants of uterovaginal prolapse in Western Ethiopia. Int Urogynecol J.

[25] Sheleme T, Mamo G, Melaku T, Sahilu T (2020). Glycemic control and its predictors among adult diabetic patients attending Mettu Karl Referral Hospital, Southwest Ethiopia: a prospective observational study. Diabetes Therapy.

[26] Teni FS, Burström K, Berg J, Leidl R, Rolfson O (2020). Predictive ability of the American Society of Anaesthesiologists physical status classification system on health-related quality of life of patients after total hip replacement: comparisons across eight EQ-5D-3L value sets. BMC Musculoskelet Disord.

[27] Amone D, Okello TR, Okot C, Kitara D, Mugabi P, Ogwang MD. Short-term outcomes of laparotomy in the two teaching hospitals of Gulu university, northern Uganda. 2020.

[28] Naidoo R, Faurie M, Oosthuizen G, Hardcastle T (2021). Comparative outcome analysis of trauma and non-trauma emergency laparotomy using a modified NELA tool format. S Afr J Surg.

[29] Gebremedhn EG, Agegnehu AF, Anderson BB (2018). Outcome assessment of emergency laparotomies and associated factors in low resource setting. A case series. Annals Med Surg.

[30] Achiek M, Tawad F, Alier B, Yur C (2015). One Stop Management of Sigmoid Volvulus in an African setting with limited resources. East Cent Afr J Surg.

[31] Hendriksen BS, Keeney L, Morrell D, Candela X, Oh J, Hollenbeak CS et al. Epidemiology and perioperative mortality of exploratory laparotomy in rural Ghana. Annals Global Health. 2020;86(1). 10.5334/aogh.2586 PMC7047759 32140429

[32] Tengberg L, Cihoric M, Foss N, Bay-Nielsen M, Gögenur I, Henriksen R (2017). Complications after emergency laparotomy beyond the immediate postoperative period–a retrospective, observational cohort study of 1139 patients. Anaesthesia.

[33] Joseph B, Zangbar B, Pandit V, Kulvatunyou N, Haider A, O’Keeffe T (2014). Mortality after trauma laparotomy in geriatric patients. J Surg Res.

[34] Harvin JA, Maxim T, Inaba K, Martinez-Aguilar MA, King DR, Choudhry AJ (2017). Mortality following emergent trauma laparotomy: a multicenter, retrospective study: mortality after emergent trauma laparotomy. J Trauma Acute care Surg.

[35] St-Louis E, Sudarshan M, Al-Habboubi M, El-Husseini Hassan M, Deckelbaum D, Razek T (2016). The outcomes of the elderly in acute care general surgery. Eur J Trauma Emerg Surg.

[36] Luedi MM, Kauf P, Mulks L, Wieferich K, Schiffer R, Doll D (2016). Implications of patient age and ASA physical status for operating room management decisions. Anesth Analgesia.

[37] Poulton TE, Moonesinghe R, Raine R, Martin P, Anderson ID, Bassett MG (2020). Socioeconomic deprivation and mortality after emergency laparotomy: an observational epidemiological study. Br J Anaesth.

[38] Aitken RM, Partridge JS, Oliver CM, Murray D, Hare S, Lockwood S (2020). Older patients undergoing emergency laparotomy: observations from the National Emergency Laparotomy Audit (NELA) years 1–4. Age Ageing.

[39] Peacock O, Bassett M, Kuryba A, Walker K, Davies E, Anderson I (2018). Thirty-day mortality in patients undergoing laparotomy for small bowel obstruction. J Br Surg.

[40] Chua MSH, Chan DKH (2020). Increased morbidity and mortality of emergency laparotomy in elderly patients. World J Surg.

[41] Hacım NA, Akbaş A, Ulgen Y, Aktokmakyan TV, Meric S, Tokocin M (2021). Association of Preoperative Risk Factors and mortality in older patients following emergency abdominal surgery: a retrospective cohort study. Annals Geriatric Med Res.

[42] Ahmed M, Garry E, Moynihan A, Rehman W, Griffin J, Buggy D (2020). Perioperative factors associated with postoperative morbidity after emergency laparotomy: a retrospective analysis in a university teaching hospital. Sci Rep.

[43] Hajibandeh S, Hajibandeh S, Shah J, Martin J, Abdelkarim M, Murali S (2021). The risk and predictors of mortality in octogenarians undergoing emergency laparotomy: a multicentre retrospective cohort study. Langenbeck’s Archives Surg.

[44] Poulton T, Murray D, team NELAp (2019). Pre-optimisation of patients undergoing emergency laparotomy: a review of best practice. Anaesthesia.

[45] Stahlschmidt A, Novelo B, Freitas LA, Passos SC, Dussán-Sarria JA, Félix EA (2018). Predictors of in-hospital mortality in patients undergoing elective surgery in a university hospital: a prospective cohort. Revista brasileira de anestesiologia.

[46] Becher RD, Peitzman AB, Sperry JL, Gallaher JR, Neff LP, Sun Y (2016). Damage control operations in non-trauma patients: defining criteria for the staged rapid source control laparotomy in emergency general surgery. World J Emerg Surg.

[47] Singer M, Deutschman CS, Seymour CW, Shankar-Hari M, Annane D, Bauer M (2016). The Third International Consensus definitions for Sepsis and septic shock (Sepsis-3). JAMA.

[48] Rhodes A, Evans LE, Alhazzani W, Levy MM, Antonelli M, Ferrer R (2017). Surviving sepsis campaign: international guidelines for management of sepsis and septic shock: 2016. Intensive Care Med.

[49] Liu V, Escobar GJ, Greene JD, Soule J, Whippy A, Angus DC (2014). Hospital deaths in patients with sepsis from 2 independent cohorts. JAMA.

[50] Baison GN. Outcomes of Laparotomy at a Large Referral Center in Rwanda 2015.

[51] Wu J, Shu P, He H, Li H, Tang Z, Sun Y et al. Predictors of mortality in patients with acute small-bowel perforation transferred to ICU after emergency surgery: a single-centre retrospective cohort study. Gastroenterol Rep. 2021. 10.1093/gastro/goab054 PMC8972993 35382163

[52] Power GS, Harrison DA (2014). Why try to predict ICU outcomes?. Curr Opin Crit Care.

[53] Ogbuanya AU-O, Ugwu NB (2021). Emergency laparotomy at district hospitals in a developing nation: a review of indications and outcomes of treatment. J Emerg Pract Trauma.

[54] Marfil-Garza BA, Belaunzarán-Zamudio PF, Gulias-Herrero A, Zuñiga AC, Caro-Vega Y, Kershenobich-Stalnikowitz D (2018). Risk factors associated with prolonged hospital length-of-stay: 18-year retrospective study of hospitalizations in a tertiary healthcare center in Mexico. PLoS ONE.

[55] Lee SY, Lee S-H, Tan JH, Foo HS, Phan PH, Kow AW (2018). Factors associated with prolonged length of stay for elective hepatobiliary and neurosurgery patients: a retrospective medical record review. BMC Health Serv Res.

[56] FF MT MAS. Prevalence and risk factors associated with post operative infections in the Limbe Regional Hospital of Cameroon. open Surg J. 2014;8(1).

